# Supplementary material for: Characterization and Preliminary Safety Evaluation of Akkermansia muciniphila PROBIO
Source: Foods. 2024 Jan 30;13(3):442. doi: 10.3390/foods13030442 (PMC10855611; doi:10.3390/foods13030442)
Supplement: Supplementary file 1 [file foods-13-00442-s001.zip › foods-2811848-supplementary.pdf]

Table S1. Two genes related to the potential drug resistance of *A. muciniphila* PROBIO detected based on the CARD database.

ORF_ID	Cut_off	Best_hit_bitscore	Best_hit_ARO	Best_identities	ARO	Drug class	Resistance mechanism	Percentage length of reference sequence
Scaffolds_7_107624_39	Strict	761.1	*adeF*	41.27	3000777	fluoroquinolone antibiotic; tetracycline antibiotic	antibiotic efflux	99.72
Scaffolds_25_1658_1	Strict	457.6	*ANT(3'')-IIa*	100	3004089	aminoglycoside antibiotic	antibiotic inactivation	70.59

Table S2. One gene related to the potential drug resistance of *A. muciniphila* PROBIO detected based on the ResFinder database.

Resistance gene	Identity	Contig or depth	Position in contig	Phenotype	Accession
aadA15	96.20	Scaffolds_25_1658_1	1..685	spectinomycin; streptomycin	DQ393783

Table S3. Genome sequence characteristics of *A. muciniphila* PROBIO.

Sequence ID	Circular	Sequence length	GC content	A content	T content	G content	C content	N content
chr	Circular	2,863,710	55.32%	642,274(22.43%)	637,142(22.25%)	788,279(27.53%)	796,015(27.80%)	0(0.00%)
Total	●	2,863,710	55.32%	642,274(22.43%)	637,142(22.25%)	788,279(27.53%)	796,015(27.80%)	0(0.00%)

Table S4. A gene related to virulence factors of *A. muciniphila* PROBIO detected based on the VFD database.

qseqid	sseqid	pident	length	e-value	bitscore	description
Scaffolds_2_441681_74	VFG046465(gb|YP_169203.1)	73.1	394	1.80E-177	616.3	(*tufA*) elongation factor Tu [EF-Tu (VF0460)] [*Francisella tularensis* subsp. *tularensis* SCHU S4]

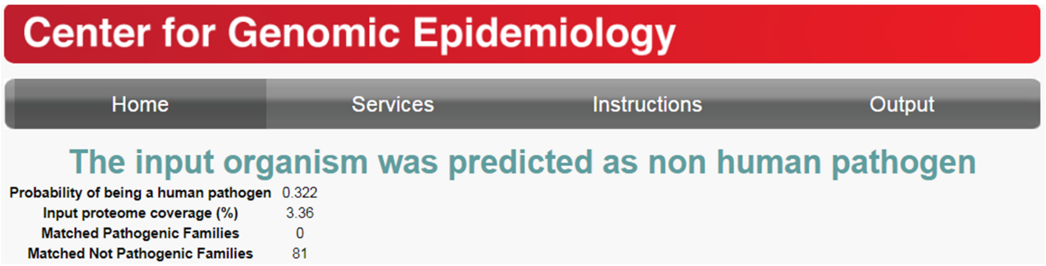

Figure S1. Prediction of the pathogenicity of *A. muciniphila* PROBIO.
